# Supplementary material for: Dynamic transcriptomic profiles of zebrafish gills in response to zinc supplementation
Source: BMC Genomics. 2010 Oct 11;11:553. doi: 10.1186/1471-2164-11-553 (PMC3091702; doi:10.1186/1471-2164-11-553)
Supplement: Additional file 2 — Interactive Direct Interaction Network representing the molecular interactions between zinc, copper, iron, calcium and proteins encoded by transcripts changed by zinc supplementation. Mini web-site containing index.html and hyperlinked pages in subdirectory describing a Direct Interaction Network automatically generated based on curated interactions contained within the proprietary PathwayArchitect database. Ovals represent proteins and the circles symbolize metal ions. Objects are coloured by their abundance in zebrafish at the time-point they were significantly different from the control is a scale from -4 fold (dark green) to +4 fold (dark red). Where significant differences were found at more than one time-point, the colour overlay shows expression at the first instance. Dark blue squares denote 'binding', and light blue squares 'expression'; green squares stand for 'regulation', green diamonds for 'metabolism', and green circles for 'promoter binding'. Arrow heads indicate directionality of the interaction where annotated. All nodes and edges can be further interrogated by selecting the relative area of the image. [file 1471-2164-11-553-S2.zip › PathwayArchitect Zn xs DIN/661635.html]

# REGULATION:

|  |  |
| --- | --- |
| Type | REGULATION |
| Effect | Positive |


---

|  |  |
| --- | --- |
| Score | 0 |


---

|  |  |
| --- | --- |
| Reference Count | 4 |


---

|  |  |
| --- | --- |
| Mechanism | Unknown |


---

|  |  |
| --- | --- |
| Reference:0 || Sentence | "I-TAC (4-73), which lacks the first three residues, has no agonistic activity but competes for the binding of I-TAC to CXCR3-bearing cells and inhibits migration and Ca(2+) changes in such cells in response to stimulation with I-TAC, IP10, and Mig." |
| PMID | 12417585 |
| Year | 2002 |
| Species | Human |
| Journal | J Biol Chem |
| RefScore | 2 |
| Source | PArchNLP |
  |
|  |  |


---

|  |  |
| --- | --- |
 Reference:1 || Sentence | "CXCR3 was also detectable by flow cytometry in cultured human mesangial cells, in which it appeared to be functionally active, as determined by the ability of its ligand, the (interferon-gamma)-inducible protein of 10 kD (IP-10) to induce intracellular Ca2+ influx." |
| PMID | 10589690 |
| Year | 1999 |
| Species | Human |
| Journal | J Am Soc Nephrol |
| RefScore | 0 |
| Source | PArchNLP |
  ||  |  |


---

|  |  |
| --- | --- |
 Reference:2 || Sentence | "Therefore, activation of NF-kappaB and mob-1 expression by supraphysiological CCK is likely mediated by both PKC activation and elevated intracellular Ca(2+)." |
| PMID | 10666030 |
| Year | 2000 |
| Species | Rat |
| Journal | Am J Physiol Cell Physiol |
| RefScore | 0 |
| Source | PArchNLP |
  ||  |  |


---

|  |  |
| --- | --- |
 Reference:3 || Sentence | "It mediates Ca2+ mobilization and chemotaxis in response to IP10 and Mig, but does not recognize the CXC-chemokines IL-8, GRO alpha, NAP-2, GCP-2." |
| PMID | 9064356 |
| Year | 1996 |
| Species | Mouse |
|  | Human |
| Journal | J Exp Med |
| RefScore | 1 |
| Source | PArchNLP |
  |


---

|  |  |
| --- | --- |
